# The Effect of Preoperative Health Education, Delivered as Animation Videos, on Postoperative Anxiety and Pain in Femoral Fractures

**DOI:** 10.3389/fpsyg.2022.881799

**Published:** 2022-05-06

**Authors:** Yuewei Wang, Xueqin Huang, Zhili Liu

**Affiliations:** ^1^Central Sterile Supply Department (CSSD), The First Affiliated Hospital of Shantou University Medical College, Shantou, China; ^2^Cardiac Care Unit (CCU), The First Affiliated Hospital of Shantou University Medical College, Shantou, China; ^3^Department of Neurology, The First Affiliated Hospital of Shantou University Medical College, Shantou, China

**Keywords:** surgical patient, animation videos, health education, anxiety, postoperative pain

## Abstract

**Objective:**

This article explores the effect of preoperative health education, in the form of animation videos, on postoperative self-reported pain levels and anxiety in femoral fractures.

**Methods:**

Ninety cases of femoral fracture were divided at random into the oral instruction group, the recorded video group, and the animation video group, with 30 cases in each group. Sociodemographic data were collected the day before surgery. Health education was then offered in one of three ways: orally, using a recorded video, or using an animation video. On days 2, 4, and 7 after surgery, the state-trait anxiety inventory (STAI) and the visual analog scale (VAS) were used to assess postoperative anxiety and pain levels, respectively, in the participants.

**Results:**

At different time points during the evaluation, total anxiety scores in the animation and recorded video groups were significantly lower than in the oral instruction group (*P* < 0.01), and the pairwise comparisons indicated statistically significant differences (*F* = 11.04, 10.06, 10.37, *P* < 0.01). However, the levels of postoperative pain in the animation and recorded video groups were not significantly different (*P* > 0.05). STAI scores in the three groups were found to have significant interactions with the measurement time (*F* = 6.74, *P* < 0.01). However, there were no apparent interactions between the VAS score and the measurement time (*F* = 1.31, *P* > 0.05) in the three groups.

**Conclusion:**

Preoperative health education with the aid of multimedia is more effective than oral instruction in lowering patients’ postoperative anxiety and pain levels. In addition, animation videos are superior to recorded videos in mitigating postoperative anxiety. Whether the two approaches differ in reducing postoperative pain in bone fractures remains to be further tested.

## Introduction

Patients scheduled to undergo surgery for a femoral fracture might experience preoperative anxiety due to a sense of uncertainty about the prognosis (Huang et al., 2005). In the case of postoperative adverse events, the patients will likely suffer from postoperative anxiety and pain (Ma et al., 2021). Pain is an experience in which psychological factors play a key role (Varallo et al., 2021). Among these, in postoperative pain, anxiety plays a significant role (Zhang et al., 2021). Therefore, health education interventions aimed at reducing anxiety could also have an impact on pain (Kuo et al., 2021). In China, the overall incidence of preoperative anxiety in adult patients ranges from 11 to 80%, Anxiety in most patients started days to hours before surgery, and 82.4% had anxiety on the day of surgery (Gong and Zhao, 2018). Surgery-related anxiety and postoperative pain may cause sleep disorders, and other psychological and physiological complications (Li et al., 2021). If any of these conditions occur, postoperative recovery will be delayed and hospitalization length may be prolonged. This also adds to the health-related financial burden for both patients and society as a whole. According to health data published in China, 15 million people suffer from surgery-related anxiety every year, and 22.50 million report postoperative pain. For every extra day spent in the hospital, the total annual increase in hospitalization expenditure will be 13.13 billion RMB (Weiser et al., 2008; National Bureau of Statistics of the People’s Republic of China, 2011; Ministry of Health of the People’s Republic of China, 2012).

Evidence suggested that preoperative health education might be effective in reducing the incidence (Ayyadhah Alanazi, 2014). Effective preoperative health education not only enables patients to acquire relevant knowledge of the disease, but also enables the patient to actively cooperate with the doctor’s treatment, which is conducive to the recovery of the disease and reduces the occurrence of postoperative complications such as anxiety and postoperative pain in surgical patients, promote recovery, shorten hospital stays, and reduce medical costs (Wong et al., 2010). Therefore, the significance and role of health education are becoming more and more important. Medical staff do this work seriously and implement effective education content according to the needs of patients. At the same time, attention should also be paid to the health education of patients. At present, the research on the effect of preoperative education on anxiety and pain after femoral fracture is not enough to support its clinical application. In view of this, to examine the impact of three different preoperative guidance on postoperative complications in surgical patients in order to provide strong evidence for clinical application. In this study, we use three preoperative health education approaches (animation video, recorded video, and oral instruction) among patients with femoral fractures and measure the effects of these methods on postoperative anxiety and pain.

## Subjects

### Inclusion Criteria

Ninety patients, hospitalized between October 2012 and June 2013 in the Department of Orthopedics of a hospital, were included in our analysis, in accordance with the following criteria: (a) having undergone surgery for femoral fracture at the Department of Orthopedics, (b) aged over 18 years, (c) no auditory and visual disabilities, (d) awake consciousness and no psychological impairments, (e) able to speak and understand Mandarin, (f) agreed not to use analgesics after surgery, and (g) willing to participate in this experiment give their informed consent. Patient exclusion criteria for the study were as follows: cases not hospitalized in the Department of Orthopedics, or not receiving surgery for the first time, or for reasons other than femoral fracture.

### Grouping

A quasi-experimental design and three-group pre–post tests were adopted, with 30 cases in each group. Data from cases that met the exclusion criteria or who asked to leave the study were eliminated. To compensate for excluded participants, another case that met the inclusion criteria was selected. The study was approved by the Medical Ethics Committee of our hospital. In this experiment, the cases were equally divided into the animation group (who were instructed by watching an animated video), the recorded video group, and the control group (instructed orally). The case information was collected by the most convenient method possible. In order to avoid interference between patients, data from the control group were collected first, followed by data from the recorded video group and, finally, from the animation group. The preoperative data collection was performed 1 day before surgery, and consent was obtained from the patients. The informed consent for the three groups is listed in Table 1.

**TABLE 1:** General data from the three groups.

Item	Oral instruction	Recorded video	Animation video	*X*^2^ or *F*-value	*P*-value
Gender				0.638	0.727
Male	19	17	16		
Female	11	13	14		
Age (years)	44.9 ± 20.34	44.9 ± 20.34	52.8 ± 22.19	1.93	0.340
Marital status				2.273	0.927
Unmarried	6	4	6		
Married	18	21	17		
Divorced	2	1	1		
Widowed	4	4	6		
Education level				4.835	0.980
Illiterate	2	2	4		
Primary school	5	6	8		
Junior high school	7	6	8		
Senior high school	7	7	4		
Associate degree	5	5	3		
Bachelor’s degree	2	3	2		
Master’s degree and above	2	1	1		
Occupation				6.433	0.924
Jobless	5	7	10		
Military, public service, education	5	2	2		
Commerce	5	4	2		
Agriculture, fishing, and animal husbandry	4	4	6		
Industry	8	9	7		
Freelance	2	3	2		
Others	1	1	1		
Source of income				6.090	0.660
On one’s own	14	18	15		
Spouse	3	2	1		
Parent	4	2	1		
Children	8	6	12		
Others	1	2	1		
Economic status				6.706	0.349
Poor	5	3	1		
Fairly well	8	12	10		
Well-off	9	11	15		
Wealthy	8	4	4		
Payment of medical expenditure				0.550	0.996
Medical insurance	18	19	20		
At one’s own expense	3	3	3		
Others	9	8	7		

## Materials and Methods

### Approaches Toward Preoperative Health Education

The pre-intervention data were collected from all three groups. The measurement time points, the form of preoperative health education, and the specific content of preoperative health education are shown in Tables 2–4, respectively. In the 10-min health education, the video time is 3.5 min, the animation group will watch the animation video for 3.5 min, and the video group will watch the recorded video for 3.5 min. The content includes: fasting after 10:00, resting early, taking a bath if the condition allows, and the next day at 7:30 a.m., the family members will arrive at the ward and go to the operating room with the patient to introduce the general steps of the operation and the operating room environment.

**TABLE 2:** Time points of measurement.

Group	Time points				
	One day before surgery	Day of surgery	Day 2 after surgery	Day 4 after surgery	Day 7 after surgery
Control	O1	X1	O2	O3	O4
Recorded video	O1	X2	O2	O3	O4
Animation video	O1	X3	O2	O3	O4

**TABLE 3:** Procedures of pre-operative health education.

Time (min)	Procedure
1	Self-introduction
2	Data collection
10	Health education in various forms
5	Discussion

**TABLE 4:** Preoperative health education program.

Program	Content
Diet management	Fasting 8 h before surgery
	No drinking 4 h before surgery Bathing
Family members	Arrive in ward at 7:30
Surgical procedure	Position, disinfection, procedure environment of OR
Post-operative complications explained	Incision pain
	Insomnia
	Anxiety
Preoperative preparation	Personal hygiene
	Premedication

### Evaluation

#### Evaluation of Anxiety Levels

The Chinese version of the state-trait anxiety inventory (STAI) was administered to the patients in the three groups. This scale can distinguish between state (temporary) and trait (stable) anxiety and applies extensively to adults with anxiety disorders (Zhang, 2005).

Questions 1–20, corresponding to state anxiety, determined how the patients felt during the recent or perioperative period and assessed such feelings as fear, tension, worry, and nervousness. Questions 21–40, corresponding to trait anxiety, measured the frequency of anxiety. The cumulative scores of the state and trait anxiety scales were calculated, and the allowable range was 20–80 points. The higher the score, the higher the level of the specified type of anxiety.

#### Evaluation of the Pain Level

Postoperative pain was measured using visual analog scale (VAS) on a horizontal 10 cm-long straight line. A score of 0 indicated no pain, while a score of 10 indicated severe pain. The higher the score, the greater the degree of postoperative pain.

The author of this scale declared that the scale might be used for free without authorization. In addition, the scales used in this paper had good validity (Reips and Funke, 2008).

#### Quality Control

To create content for the preoperative health education methods, patients, operating room medical staff, and ward employees were interviewed before surgery and asked what subject matter should be included. These interview results were integrated with the experience of the researchers and their knowledge of evidence-based medicine. A pilot experiment was carried out using the produced video program, 10 patients with femoral fracture were selected to watch the video, and the length of the video was adjusted according to the experimental results of the patients to make it more concise and suitable for repeated viewing. Patient sampling was carried out using conventional methods, and patient groups were restricted from conferring with each other. Patients within groups were encouraged to learn from each other, and patients were rewarded with a small gift to increase their level of attention and participation in the study.

### Statistical Processing

Data were statistically analyzed using SPSS 20.0 software. The measurement data were expressed as $\bar{X}\pm S$. The data of the three groups before and after surgery were compared using repeated-measures analysis of variance. Enumeration data were analyzed by the Chi-square test or Fisher’s exact test. *P* < 0.05 was considered statistically significant.

## Results

### Anxiety Levels of the Three Groups at Different Time Points

**FIGURE 1:**
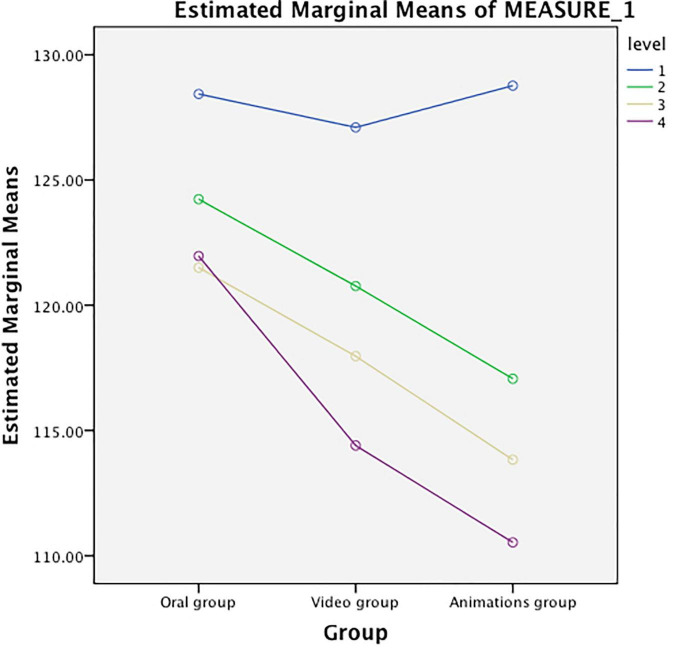
Intergroup differences in the anxiety levels between the three groups.

As seen from Table 5 and Figure 1, the preoperative STAI scores were not significantly different among the three groups (*P* > 0.05). All the STAI scores in the animation video group were much lower than those of the recorded video group on days 2, 4, and 7 after surgery. The STAI scores of the latter were markedly lower than those of the oral instruction group (*P* < 0.01). The STAI scores of the three groups varied in different ways throughout the duration of the study, which suggested an interaction with the factor of time (*F* = 6.74, *P* < 0.01, Table 6). The anxiety level was lowest in the animation video group and highest in the control group.

**TABLE 5:** Anxiety levels in the three groups.

Group	*N*	Before surgery	After surgery			*F*-value	*P*-value
			Day 2 after surgery	Day 4 after surgery	Day 7 after surgery		
Oral health education	30	128.43 ± 6.18	124.23 ± 6.21	121.50 ± 6.83	121.97 ± 15.44	3.325	0.022
Recorded video	30	127.10 ± 6.07	120.77 ± 5.89	117.97 ± 6.14	114.40 ± 5.34	25.09	0.000
Animation video	30	128.77 ± 6.27	117.07 ± 7.12	113.83 ± 6.87	110.53 ± 5.17	46.054	0.000
*F*-value		0.611	9.321	10.059	10.361		
*P*-value		0.545	0.000	0.000	0.000		

**TABLE 6:** Interaction between anxiety level and time.

	Sum of squares	Degree of freedom	Mean square	*F*-value	*P*-value
Anxiety level × time	1165.050	6	194.175	6.740	0.000

### Pain Levels in the Three Groups at Different Time Points

**FIGURE 2:**
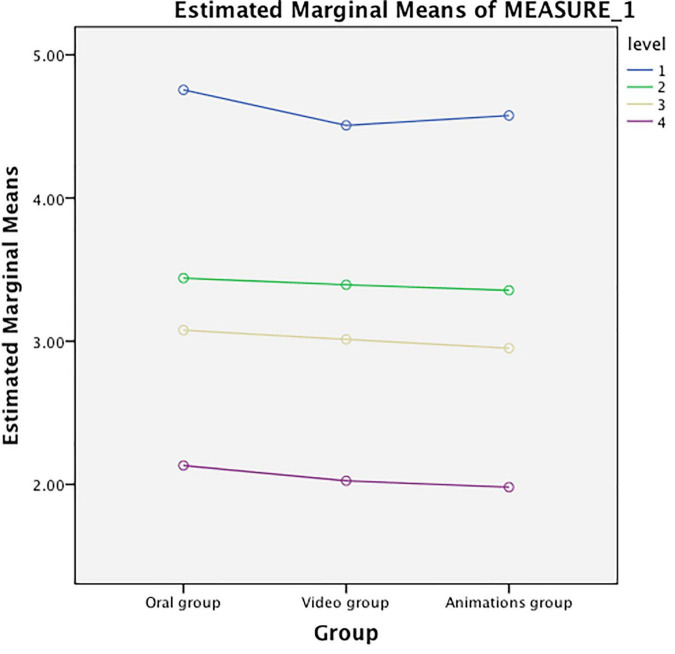
Intergroup differences in the pain levels between the three groups.

As shown in Table 7 and Figure 2, the three groups did not differ significantly from each other in terms of the VAS scores before surgery (*P* > 0.05). On days 2, 4, and 7 after surgery, the VAS scores in the animation video group showed no distinct differences from those in the recorded video group (*P* > 0.05). In these two groups, the VAS scores were considerably lower than those in the oral instruction group (*P* < 0.01). The trend variations in the VAS scores in the three groups were consistent over time, indicating no apparent interaction with the factor of time (*F* = 1.31, *P* > 0.05), as seen in Table 8. Pain levels in the animation video group and the recorded video group were lower compared with the oral instruction group.

**TABLE 7:** Pain levels in the three groups.

Group	*n*	Before surgery	After surgery			*F*-value	*P*-value
			Day 2 after surgery	Day 4 after surgery	Day 7 after surgery		
Oral instruction group	30	4.76 ± 0.51	3.44 ± 0.06	3.07 ± 0.06	2.13 ± 0.09	515.282	0.000
Recorded video group	30	4.51 ± 0.47	3.39 ± 0.10	3.01 ± 0.09	2.02 ± 0.07	570.455	0.000
Animation video group	30	4.57 ± 0.49	3.35 ± 0.10	2.95 ± 0.11	1.98 ± 0.18	463.442	0.000
*F*-value		2.095	7.284	14.226	12.367		
*P*-value		0.129	0.001	0.000	0.000		

**TABLE 8:** Interaction between pain level and time.

	Sum of squares	Degree of freedom	Mean square	*F*-value	*P*-value
Pain level × time	0.404	6	0.067	1.309	0.253

## Discussion

### The Importance of Preoperative Health Education

Health knowledge and information are the basis for establishing positive health beliefs and attitudes and subsequent health behaviors. Health education is of great significance to patients since it not only helps them to understand their condition and take appropriate health behaviors to promote treatment and rehabilitation but also reduces fear and anxiety caused by the unknown.

Anxiety is the experience of an adverse emotional reaction to a potential threat, whereas fear leads to avoidance behavior, whereas anxiety is associated with uncontrollable or inevitable events (Michael et al., 2000). Surgery-related anxiety is very prevalent among patients, regardless of the type of surgery to be performed. However, the degree of anxiety affects the coping ability of surgical patients during the perioperative period, as they are prone to worrying about possible complications, recovery, and adverse events following surgery (Eijlers et al., 2019; Kuner and Kuner, 2021).

Surgery can cause damage to the body and can cause a sympathetic nervous system response. Tissue damage triggers the production of histamine and other inflammatory cytokines, and nociceptors are stimulated and produce pain signals that are transmitted to the brain, producing postoperative pain (Alattas et al., 2017). About 80% of surgical patients worldwide suffer from severe postoperative pain, which, if left untreated, will affect their quality of sleep and impede postoperative recovery. As a consequence, hospitalization will be prolonged (Alattas et al., 2017). This not only increases the economic burden for patients and society but also lowers patient satisfaction (Rabah et al., 2020; Small and Laycock, 2020; Dieu et al., 2021; Michael, 2021; Moghalu et al., 2021).

Preoperative health education is highly important for all surgical patients. Steves and Scafide (2021) found that preoperative health education shortened the hospital stay and saved on medical expenditure. Salibian et al. (2021) showed that the levels of anxiety and postoperative pain in women with breast cancer were much lower after the implementation of preoperative health education. Research by Wong et al. (2010) also indicated that patients benefited from health education, as pain and anxiety levels were lowered and self-efficacy was improved. Gholami et al. (2020), Sorel et al. (2020), and Rai et al. (2021) demonstrated that preoperative health education, when fully individualized and carried out correctly, can significantly reduce the level of postoperative anxiety and pain in surgical patients.

### Importance of Preoperative Health Education in the Form of an Animation Video

As a measure to boost postoperative recovery, preoperative health education may be traditionally performed by oral instruction or group teaching, and it can rely on two-dimensional printed materials or multimedia. The traditional form of preoperative education, due to its common form and small impact, cannot achieve the effect expected by doctors. Recorded videos are often overloaded with educational content, and scenes depicting surgical bleeding and tissue incision may aggravate anxiety. Animation videos allow a lively representation of the contents of health education, which systematically instructs patients and their relatives on the surgery that they are going to undergo and the matters that require attention. If necessary, patients can watch the video repeatedly before surgery, and the risk of cross-infection caused by distributing and reading pamphlets does not exist. The use of videos for preoperative health education also reduces the workload of healthcare workers and its associated costs, as they otherwise would have to go from bed to bed to instruct patients orally.

With advances in multimedia technology, modes of health education in hospitals are constantly changing, and many medical organizations in China have carried out multimedia video-based health education activities (Yang et al., 2021). Animated health education videos based on multimedia broadcasting mitigate faults in traditional oral and educational manuals. With the help of rich and easy-to-understand animated videos, patients and their families can increase their understanding of health-related content through both sound and images, which meets the learning needs of patients of different ages and educational levels. Educational videos can be watched repeatedly so that patients may master increased knowledge. In addition, through animation video health education, and the joint efforts of relevant health educators in various nursing units, the hospital’s nursing level can be improved.

### The Role of Preoperative Health Education, in the Form of Animation Videos, in Alleviating Postoperative Anxiety and Pain

In recent years, medical disputes caused by insufficient health education have occurred frequently, and, therefore, the provision of adequate health education plays an important role in medicine today. Animation videos can be applied as a useful tool, not only to promote a better and transparent doctors-patients relationship but also to reduce preoperative anxiety among children and adults (Michalski et al., 2016; Platto et al., 2019; Nair et al., 2021).

In this study, it was found that postoperative anxiety levels in patients with femoral fractures were lowered considerably after watching an animation video. The smallest reduction in anxiety was found with oral instruction (*P* < 0.01). The STAI score was significantly lower in the early period after surgery (day 2) in the animation video group than in the other two groups (*P* < 0.01). At each time point after surgery, the STAI scores of all three groups significantly decreased compared with those before surgery (*P* < 0.01). This suggests that the anxiety levels of patients declined rapidly with time following sufficient health education. The pain levels of the three groups varied consistently over time. The animation video group and recorded video group showed significant differences in the effectiveness of alleviating pain compared with the oral instruction group as the control (*P* < 0.01), the difference between the animation group and the oral information group is that the difference before and after watching the animation video group is greater than the difference before and after oral instruction group, but the two experimental groups did not differ significantly (*P* > 0.05). Although all three approaches toward preoperative health education were effective to some extent, the animation video provided the greatest benefit in alleviating either postoperative anxiety or postoperative pain. However, the use of an animation video did not achieve a significantly prominent effect in pain relief compared with the recorded video. The reason may be the similarity between the content taught by the two types of media and the fact that the recorded video contains few elements that may affect patient pain levels. The patients in our group generally agreed on the importance of animation videos as a preoperative health education tool, and therefore this method deserves further investigation.

In addition to enhancing patient understanding, animation videos convey additional benefits over other forms of preoperative health education, such as oral instruction and printed materials. For example, the method of playing the video can be adapted according to the specific conditions, such as the hardware facilities available at the hospital. The contents of the video to be played can be determined by the patients themselves or by their relatives, depending on their learning ability and individual needs. Thus, multimedia health education using animation videos meets the demands of a more diverse group of patients and achieves higher learning effectiveness. In addition, the patients and their relatives are offered a complete set of health education programs suitable for repeated viewing.

To sum up, preoperative health education in the form of animation videos can reduce the levels of postoperative anxiety and pain in patients with femoral fractures. Compared with the recorded video and oral instruction groups, the use of animation videos facilitated knowledge assimilation related to surgery and recovery, strengthened patients’ coping abilities, and lowered postoperative anxiety and pain levels. Therefore, it is worth popularizing this approach to preoperative health education in medical practices. However, since this study is affected by limitations, such as a small sample size and a lack of randomization, further investigations are required to confirm the conclusions reached in this paper.

## Data Availability Statement

The original contributions presented in the study are included in the article/supplementary material, further inquiries can be directed to the corresponding author.

## Ethics Statement

The studies involving human participants were reviewed and approved by the First Affiliated Hospital of Shantou University Medical College. The patients/participants provided their written informed consent to participate in this study.

## Author Contributions

YW conceived of the study. XH participated in its design and coordination. ZL helped to draft the manuscript. All authors read and approved the final manuscript.

## Conflict of Interest

The authors declare that the research was conducted in the absence of any commercial or financial relationships that could be construed as a potential conflict of interest.

## Publisher’s Note

All claims expressed in this article are solely those of the authors and do not necessarily represent those of their affiliated organizations, or those of the publisher, the editors and the reviewers. Any product that may be evaluated in this article, or claim that may be made by its manufacturer, is not guaranteed or endorsed by the publisher.
